# The Aim2Be mHealth Intervention for Children With Overweight or Obesity and Their Parents: Person-Centered Analyses to Uncover Digital Phenotypes

**DOI:** 10.2196/35285

**Published:** 2022-06-22

**Authors:** Olivia De-Jongh González, Claire N Tugault-Lafleur, E Jean Buckler, Jill Hamilton, Josephine Ho, Annick Buchholz, Katherine M Morrison, Geoff DC Ball, Louise C Mâsse

**Affiliations:** 1 School of Population and Public Health, University of British Columbia BC Children's Hospital Research Institute Vancouver, BC Canada; 2 School of Nutrition Sciences, Faculty of Health Sciences, The University of Ottawa. Ottawa, ON Canada; 3 School of Exercise Science, Physical and Health Education, University of Victoria. Victoria, BC Canada; 4 Department of Paediatrics Hospital for Sick Children University of Toronto Toronto, ON Canada; 5 Cumming School of Medicine, Department of Pediatrics, University of Calgary Calgary, AB Canada; 6 Children’s Hospital of Eastern Ontario Research Institute Ottawa, ON Canada; 7 Department of Pediatrics, Center for Metabolism, Obesity and Diabetes Research, McMaster University Hamilton, ON Canada; 8 Department of Pediatrics, Faculty of Medicine & Dentistry, University of Alberta Edmonton, AB Canada

**Keywords:** mobile health, mHealth, childhood obesity, digital phenotypes, latent class analysis

## Abstract

**Background:**

Despite the growing number of mobile health (mHealth) interventions targeting childhood obesity, few studies have characterized user typologies derived from individuals’ patterns of interactions with specific app features (digital phenotypes).

**Objective:**

This study aims to identify digital phenotypes among 214 parent-child dyads who used the Aim2Be mHealth app as part of a randomized controlled trial conducted between 2019 and 2020, and explores whether participants’ characteristics and health outcomes differed across phenotypes.

**Methods:**

Latent class analysis was used to identify distinct parent and child phenotypes based on their use of the app’s behavioral, gamified, and social features over 3 months. Multinomial logistic regression models were used to assess whether the phenotypes differed by demographic characteristics. Covariate-adjusted mixed-effect models evaluated changes in BMI *z* scores (*z*BMI), diet, physical activity, and screen time across phenotypes.

**Results:**

Among parents, 5 digital phenotypes were identified: *socially engaged* (35/214, 16.3%), *independently engaged* (18/214, 8.4%) (*socially* and *independently engaged* parents are those who used mainly the social or the behavioral features of the app, respectively), *fully engaged* (26/214, 12.1%), *partially engaged* (32/214, 15%), and *unengaged* (103/214, 48.1%) users. Married parents were more likely to be *fully engaged* than *independently engaged* (*P*=.02) or *unengaged* (*P*=.01) users. *Socially engaged* parents were older than *fully engaged* (*P*=.02) and *unengaged* (*P*=.01) parents. The latent class analysis revealed 4 phenotypes among children: *fully engaged* (32/214, 15%), *partially engaged* (61/214, 28.5%), *dabblers* (42/214, 19.6%), and *unengaged* (79/214, 36.9%) users. *Fully engaged* children were younger than *dabblers* (*P*=.04) and *unengaged* (*P=*.003) children. *Dabblers* lived in higher-income households than *fully* and *partially engaged* children (*P*=.03 and *P*=.047, respectively). *Fully engaged* children were more likely to have *fully engaged* (*P*<.001) and *partially engaged* (*P*<.001) parents than *unengaged* children. Compared with *unengaged* children, *fully* and *partially engaged* children had decreased total sugar (*P*=.006 and *P*=.004, respectively) and energy intake (*P*=.03 and *P*=.04, respectively) after 3 months of app use. *Partially engaged* children also had decreased sugary beverage intake compared with *unengaged* children (*P*=.03). Similarly, children with *fully engaged* parents had decreased *z*BMI, whereas children with *unengaged* parents had increased *z*BMI over time (*P*=.005). Finally, children with *independently engaged* parents had decreased caloric intake, whereas children with *unengaged* parents had increased caloric intake over time (*P*=.02).

**Conclusions:**

Full parent-child engagement is critical for the success of mHealth interventions. Further research is needed to understand program design elements that can affect participants’ engagement in supporting behavior change.

**Trial Registration:**

ClinicalTrials.gov NCT03651284; https://clinicaltrials.gov/ct2/show/NCT03651284

**International Registered Report Identifier (IRRID):**

RR2-10.1186/s13063-020-4080-2

## Introduction

### Background

Childhood obesity remains a significant health problem in Canada [1]. Evidence shows that family-based multicomponent interventions that integrate self-regulatory strategies (ie, goal setting, graded tasks, and self-monitoring) and support changes at the familial and individual levels are necessary to significantly affect child weight outcomes (eg, BMI, waist to hip ratio, and total fat mass [2-9]). However, a 2018 meta-analysis [4] found that family-based multicomponent behavioral interventions had a small effect in reducing children’s BMI in efficacy trials versus standard-of-care controls (β=−.16, 95% CI −0.24 to −0.07).

Mobile health (mHealth) interventions offer a promising adjunct or alternative to in-person treatments to support lifestyle behavior change [10,11]. Several reviews [12-15] and meta-analyses [16,17] have suggested that mHealth interventions offer multiple advantages to in-person interventions (eg, real-time data collection, intervention in natural environments, lower costs, health behavior tracking with feedback, and incorporation of gamified elements), which may appeal to children and youth [12]. Data on the efficacy of mHealth interventions for the prevention and management of childhood obesity are promising but limited as this is still a rapidly evolving field of research [12,14,18].

mHealth interventions for children living with obesity are most often evaluated using randomized controlled trials and, in some cases, evaluate the *dose* of the intervention received to provide a better understanding of their effects [19-24]. Dose-response analyses are often measured in terms of total minutes or percentage of content examined; however, this approach does not provide a nuanced picture of how users may benefit from different mHealth intervention components (ie, what design elements of the app may be more successful in engaging participants and promoting health behavior change) [25,26]. Studies examining how intervention exposure affects behavior change cannot solely focus on the quantity of the intervention received, but must also consider how participants engage with the *active ingredients* of the intervention—namely, the features that support behavior change.

mHealth interventions are particularly well-suited to examine in greater detail which components of the intervention participants engage with through app analytics data. Recently, there have been calls to develop analytical methods to process the vast amounts of data that are available when using mHealth technologies [27] and identify *digital phenotypes* (ie, user typologies derived from individuals’ patterns of interactions with specific app features) [28,29]. Although digital phenotypes have been used in other areas of health research (eg, diabetes [30], sleep [31], mental health [32]) and dietary and physical activity behaviors in a nonclinical sample [33], little attention has been paid to the treatment of obesity in childhood. Some studies have investigated which app features participants use [23,33] and individual characteristics associated with partial or total use of an intervention [33-36]. However, most studies evaluated usability derived from self-reported measures (eg, asking participants about their preferences and use of app features), total app use, or the use of individual features instead of focusing on patterns of app use [23,34,37].

### Objectives

To our knowledge, no study targeting childhood obesity has identified user typologies based on participants’ engagement with objectively measured components of an mHealth intervention. To address this gap, this study aimed to (1) identify digital phenotypes of Canadian children with overweight or obesity and their parents who used an mHealth app (the Aim2Be app [25]) over a 3-month period, (2) explore whether participants’ characteristics differed by digital phenotype, and (3) evaluate 3-month changes in children’s BMI *z* scores (*z*BMI) and dietary, physical activity, and screen time behaviors across digital phenotypes.

## Methods

### Study Design

This study was a secondary analysis of data collected from a randomized controlled trial evaluating the efficacy of the Aim2Be app (version 2) to improve lifestyle behaviors and adiposity among children with overweight or obesity [25,38]. The trial was registered on ClinicalTrials.gov (NCT03651284) on August 29, 2018 [25]. The CONSORT-EHEALTH (Consolidated Standards of Reporting Trials of Electronic and Mobile Health Applications and Online Telehealth) checklist [39] is available in Multimedia Appendix 1. Data analyzed in this study were collected from March 2019 to June 2020.

### Ethics Approval

The evaluation protocol was approved by the Children’s and Women’s Research Ethics Board at the University of British Columbia (H16-03090/H17-02032), the Health Research Ethics Board at the University of Alberta (Pro00076869), the Hospital for Sick Children Research Ethics Board (REB1000059362), the Hamilton Integrated Research Ethics Board (project 4250), and the Children’s Hospital of Eastern Ontario Research Ethics Board (18/01E). All the participants provided web-based consent before participating in the study.

### Data Collection Protocol

**Figure 1:**
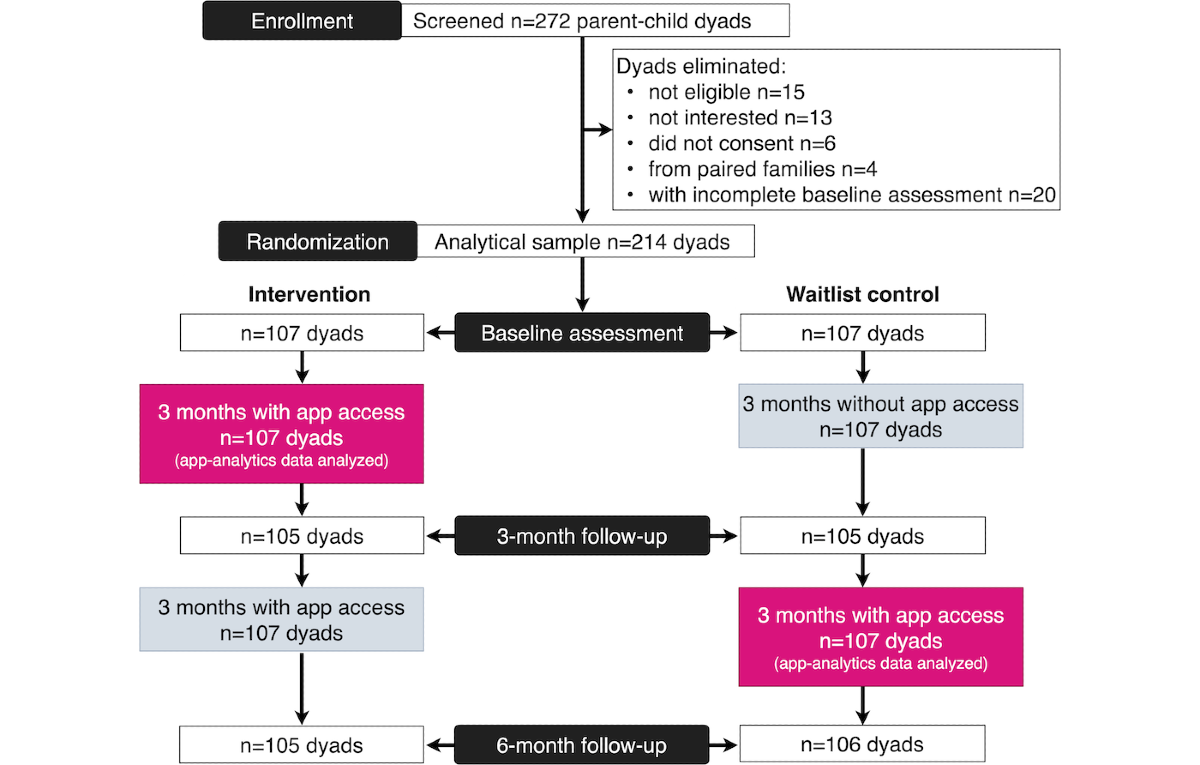
CONSORT (Consolidated Standards of Reporting Trials) flow diagram depicting the study methodology and data analyzed.

The detailed study protocol has been published elsewhere [25]. The participating families (N=214) were recruited from 6 weight management clinic sites across Canada, as well as through Facebook. Children were eligible to participate if they were aged 10 to 17 years and were overweight or obese, as defined by the age- and sex-specific World Health Organization cutoffs [40]. After providing consent, eligible participants completed a web-based survey, three 24-hour dietary recalls, and received assessment tools for height (measuring tape) weight (digital scale), and physical activity (Fitbit Flex 2, Fitbit Inc) to complete baseline measurements. Participants completed follow-up assessments at the 3- and 6-month follow-ups. Families randomized to the experimental group (107/214, 50%) had access to the app after completing baseline measures. Waitlisted control families (107/214, 50%) were given access to the app after completing their assessment at the 3-month follow-up. This study combined data collected from baseline to 3 months in the intervention group, and from 3 to 6 months in the waitlisted control group (Figure 1). Randomization was successful, and participants’ characteristics did not differ between the intervention and the waitlisted control group; however, our analyses were not based on the randomization group but dependent on users’ engagement.

### The Aim2Be Intervention

The theoretical foundations of the Aim2Be app have been described elsewhere [25]. Briefly, the app, which was cocreated by Ayogo Health Inc [41] and the Childhood Obesity Foundation with expert input, aimed to promote healthy lifestyle behaviors among children and their parents by targeting dietary, physical activity, screen time, and sleep behaviors while emphasizing healthy body image, strong self-esteem, and living green [25]. The behavior change techniques incorporated in the app are grounded in social cognitive theory [42], the Player Experience and Need Satisfaction Model—an extension of the self-determination theory [43,44]—and the Agency, Challenge, Uncertainty, Discovery, and Outcomes framework [45]. The content within different features of the app was also informed by the Canadian 24-Hour Movement Guidelines [46] and the Canadian dietary guidelines in place at the time of the study (Canada’s Food Guide 2007) [47].

**Figure 2:**
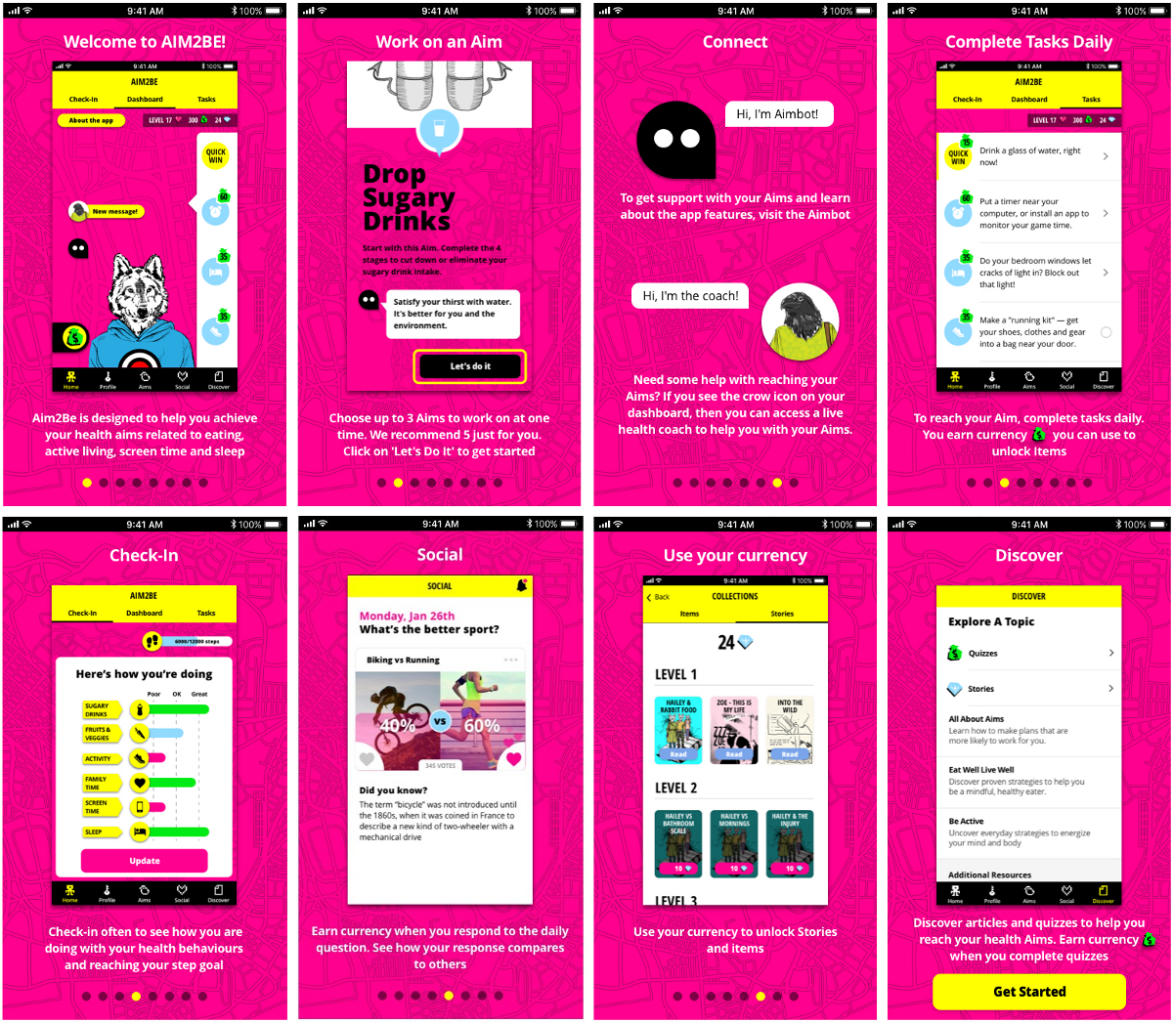
Screenshot of the Aim2Be app for children.

**Figure 3:**
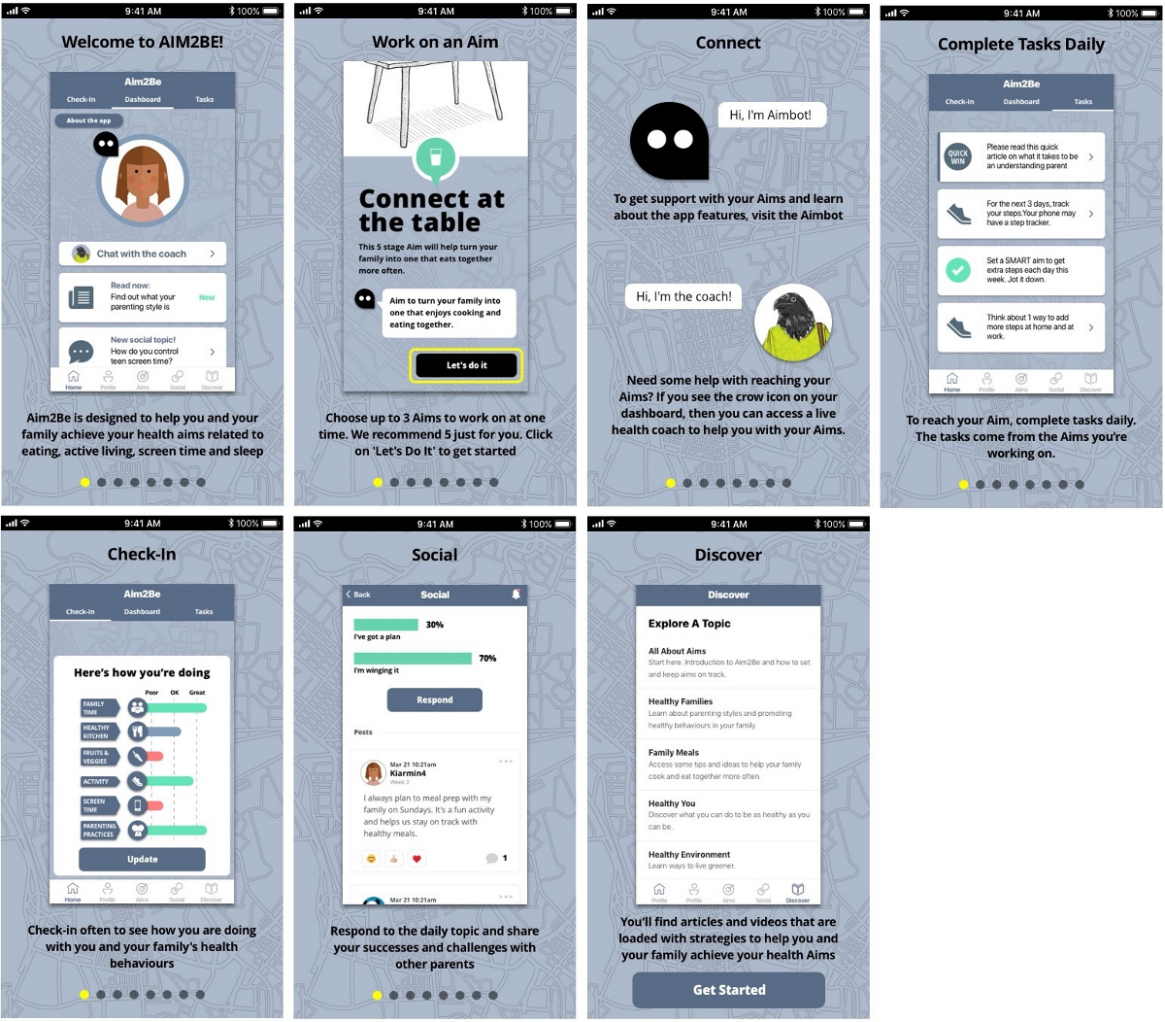
Screenshot of the Aim2Be app for parents.

The Aim2Be app features fall under 3 broad domains: behavioral, gamified, and social. The behavioral domain draws on self-regulatory strategies such as goal setting, self-monitoring, and graded tasks to facilitate behavior change by strengthening self-regulatory skills [4,8]. The gamified domain focuses on increasing participants’ enjoyment, engagement, and motivation through various gamification elements (eg, personalization, challenges, uncertainty). The social domain facilitates peer support, behavior modeling, and interaction with other app users or with a coach through different interactive features (eg, answering poll questions, viewing poll results, posting on the social wall, and responding to others’ posts). Social support is also provided to children through a companion app for parents, which aims to facilitate behavioral changes through a positive familial environment, reinforcement strategies, and environmental and stimulus control. Figures 2 and 3 illustrate screenshots of the child and parent interventions, respectively.

In addition to the parent companion app, 2 very similar versions of Aim2Be were developed for preteens (aged 10-13 years) and teenagers (aged 13-17 years), with 3 app features (ie, stages, posting on a social wall, and responding to others’ posts) available only to teenagers. As this study combined data from both teenagers and preteens, features only available to both groups were included in the analyses.

### Measures

#### Use of Aim2Be App Features

App analytics (provided by Ayogo Health Inc [41]) were used to track the number of times children and parents used each Aim2Be app feature. The behavioral domain included the following five features: (1) *aims*, indicating the number of high-level goals chosen by users while indicating their perceived importance and potential obstacles; (2) *tasks*, indicating the number of activities users completed to accomplish their aims; (3) *check-ins*, indicating the number of times users self-monitored their progress regarding specific health behaviors, with short recommendations on how to improve their behaviors; (4) *articles read*, indicating the number of articles providing educational content read by the user; and (5) *articles reflected on*, indicating the number of written responses provided by the user after reading an article.

The gamified domain included the following four features: (1) *quick wins*, which involved simple tasks users completed to engage in a healthy behavior or explore a new feature of the app, and which allowed them to earn digital currency; (2) *stories*, which involved the number of times users read *choose your own adventure* stories (these stories used a user-guided fictional character involved in a series of decision-making processes); (3) *quizzes*, which involved short tests that the users answered, allowing them to earn digital currency if they selected the correct answer; and (4) *collections*, which involved digital items the user purchased with digital currency within the app. The parent app integrated only *quick wins* as the gamified domain.

The social domain included the following four features: (1) *answer poll*, which involved the number of 2-choice poll questions from the social poll responded to by the users, with feedback on the percentage of users who selected each option; (2) *digital coach*, which involved the number of chat sessions between the user and a digital coach with preprogrammed prompts, questions, and answers; (3) *live coach*, which involved the number of one-on-one messages the user sent to a live trained health coach; and (4) *posts*, which involved the number of times the user posted a message on a social wall, sharing thoughts, feelings, or experiences with others. By design, the live coach feature, analyzed as part of the social domain, was not made available to participants randomized to the waitlisted control group; therefore, data for these participants were missing at random. In addition, only teens (119/214, 55.6%) had access to the posting feature. Consequently, this feature was not analyzed in the children’s sample as the missing data were age-related.

#### Children’s zBMI

Parents were mailed a digital scale (Active Era) and a measuring tape (HDX Corp) with instructions (using the Centers for Disease Control and Prevention home protocol [48]) to accurately measure their child’s height and weight at home. This procedure has been validated to assess children’s height and weight at home [49]. Children’s height and weight were then used to compute *z*BMI using the World Health Organization Stata macro [40], where being overweight in childhood was defined as *z*BMI >1 SD and ≤2 SD, and obesity was defined as *z*BMI >2 SD.

#### Health Behaviors

##### Dietary Behaviors

Children’s dietary behaviors were evaluated with the Waterloo Eating Behavior Questionnaire, a 24-hour web-based dietary recall (intraclass correlation coefficients ranging from 0.39 to 0.71 for energy, carbohydrates, sugar, fiber, and fats, validated against dietitian interviews) [50]. Children reported all foods and beverages consumed on the previous day. Standardized food group servings using the 2007 Canada’s Food Guide classification framework were used to quantify the amount of food consumed (eg, number of servings of vegetables and fruits) [47]. A composite index of dietary quality (the Canadian Healthy Eating Index [51]) was used as a measure of overall adherence to the 2007 Canada’s Food Guide. The index ranges from 0 to 100 points, where scores <50, 50 to 80, and >80 indicate *poor*, *requiring improvement*, and *good* dietary quality, respectively [51]. Parents’ dietary behaviors were evaluated using 7 items adapted from the Canadian Community Health Surveys [52]. Parents reported their own consumption of vegetables and fruits (excluding fruit juices), 100% fruit juices, and sugar-sweetened beverages.

##### Physical Activity

Children’s physical activity was evaluated using Fitbit Flex 2 (Fitbit Inc). Children wore the Fitbit for 7 to 14 days at baseline and at 3 and 6 months, and their daily step count was obtained by our team using Fitabase, a web-based platform designed for research using Fitbits [53]. Furthermore, we computed the children’s average number of daily steps. In addition, children completed a web-based survey, which included 5 questions from the Physical Activity Questionnaire for Older Children [54]; a 7-day recall inquiring about the amount of physical activity with responses between *none* and *more than 2 hours*. The total score of the questionnaire was significantly related to moderate and vigorous physical activity using accelerometers (*r*=0.33) [55]. Parents’ physical activity was evaluated using 7 items from the Physical Activity Questionnaire Short Form (repeatability reliability across different countries ranged from 0.32 to 0.88, with 75% of the correlation coefficients >0.65 and a pooled coefficient of 0.76 [56]). Participants were asked about the number of days and minutes spent sitting, walking, and engaging in vigorous and moderate physical activity over the past 7 days. The average daily time was calculated for sitting, walking, and moderate and vigorous physical activity.

##### Screen Time

Children’s and parents’ screen time was evaluated with 2 items adapted from the Sedentary Behavior Questionnaire for adults (intraclass correlation coefficient ranged from 0.51 to 0.93 [57]). Children and parents reported the time (minutes) spent watching television; playing computer or video games; using a computer, tablet, or mobile device outside of school or paid work; and talking or texting on a cell phone during their most recent week and weekend day. The average daily sedentary time was then calculated.

#### Statistical Approach

Latent class analysis (LCA) in MPlus version 8 (Muthen and Muthen [58]) was used to separately identify digital phenotypes among children and parents. There is no fixed minimum sample size for LCA as it depends on multiple factors (eg, number and quality of indicators, class differentiation, and relative samples in each class) [59]. Of relevance, previous Monte Carlo simulations [60] have found that an LCA with 100 participants can result in reliable solutions when conducted with robust indicators, providing support for conducting an LCA with 214 participants. The LCA identified digital phenotypes based on different use patterns for the various behavioral, social, and gamified app features, similar to a recent study profiling child users using an earlier version of Aim2Be [33]. As the distribution of use for each feature was skewed, an individual’s use of each app feature was ranked as *no use* (a participant never used a given app feature), and among the remaining participants, *low use* (a participant’s use of a feature was at or below the median use), or *high use* (a participant’s use of a feature was above the median use). The robust maximum likelihood estimator with the expectation-maximization algorithm and 2000 random starts was used. The LCA used full information maximum likelihood to handle data missing at random in the *live coach* feature (no other variables included in the LCA had missing data). Various fit and relative indices were used to compare different *k*-class solutions to determine the best number of classes to be retained with the LCA [59]. We first evaluated the Bayesian information criterion, sample size–adjusted Bayesian information criterion, Akaike information criterion, consistent Akaike information criterion, and approximate weight of evidence. For these indices, both a lower value and a meaningful decrease after adding another class to the solution are desirable. Second, we compared neighboring solutions of *k* classes (eg, 3 vs 4 classes) with the relative indices of the Vuong-Lo-Mendell-Rubin likelihood ratio test, bootstrap likelihood ratio test, and Bayes factor. For the Vuong-Lo-Mendell-Rubin and bootstrap likelihood ratio test, a significant *P* value indicates a better fit of *k* classes than with the previous model (*k*-1 classes). For the Bayes factor, higher scores indicate stronger evidence supporting *k* classes than those supporting *k*+1 classes. Third, we estimated how each model was corrected by all models using the correct model probability index, where higher values are desirable. Other indicators of well-differentiated classes such as entropy and average posterior probability were also evaluated, where desirable values were >0.8% and >70%, respectively. Finally, the *k*-class solution selected also considered practical and theoretical interpretability and the relative sample size of each class. Although some authors retained classes that encompassed at least 5% of the sample [59], the authors recognized the limitations of estimating classes with a low relative prevalence (1%-8%). This was accounted for when selecting the final solution.

Multiple multinomial logistic regression models were used to evaluate the associations between digital phenotypes included as the dependent variable and demographic factors (children’s and parents’ age and sex, parental educational attainment, marital status, household income, ethnicity, and recruitment site) as independent variables. Parental phenotypes were also added as predictors of children’s phenotypes.

Mixed-effect models evaluated changes in health behaviors and *z*BMI across children’s and parents’ phenotypes. One model was run for each outcome (children’s *z*BMI, children’s and parents’ diet, physical activity, and screen time). All models included an interaction term between time and phenotype and were adjusted for children’s and parents’ age and sex, parental educational attainment, marital status, household income, ethnicity, and recruitment site. Postestimation contrasts of marginal linear predictions tested overall group differences. For outcomes with borderline significance (*P<*.10) or significant (*P<*.05) overall group differences, we conducted pairwise comparisons and calculated the Cohen effect size as follows:


f^2^ = (R^2^_AB_ – R^2^_A_) / (1 – R^2^_AB_)

Here, *B* is the predictor of interest (eg, interaction phenotype 1×time), *A* is the set of all other predictors (ie, demographics, time, and other phenotypes), *R*^2^_AB_ is the proportion of variance that *A* and *B* together (ie, the full model) account for, and *R*^2^_A_ is the proportion of variance the predictors explain in a reduced model, with all fixed effects from the full model, except for the effect of *B* and random effects constrained to be the same as those from the full model. Therefore, *f*^2^ represents the proportion of variance uniquely accounted for by *B* [61,62].

All regression analyses were performed using Stata (version 15; StataCorp) [63]. The significance level was set at *P*<.05.

## Results

### Demographic Characteristics of the Participants

From the 214 parent-child dyads, the mean age of the children was 13 (SD 2.2) years, and the sample was evenly split among boys (104/214, 48.6%) and girls (110/214, 51.4%). Approximately 92.5% (198/214) of the participating parents were mothers, and 71% (152/214) were married or living with a partner. The mean age of parents was 44 (SD 6.2) years. Just over half of the parents (120/214, 56.1%) had not completed a university degree. Approximately 60.3% (129/214) of parents self-identified as having a White or European descent, 16.8% (36/214) reported mixed ethnicity, 5.6% (12/214) reported an East or Southeast Asian descent, 4.2% (9/214) reported a South Asian descent, and 3.3% (7/214) reported an indigenous descent. Household income ranged from <CAD $50,000 (US $37,500; 36/214, 16.8%) to >CAD $150,000 (US $112,500; 35/214, 16.4%). Approximately 30.8% (66/214) of parents reported incomes between CAD $50,000 (US $37,500) and CAD $100,000 (US $75,000), and 25.7% (55/214) reported incomes between CAD $100,000 (US $75,000) and CAD $150,000 (US $112,500).

### Identifying Digital Phenotypes

**Figure 4:**
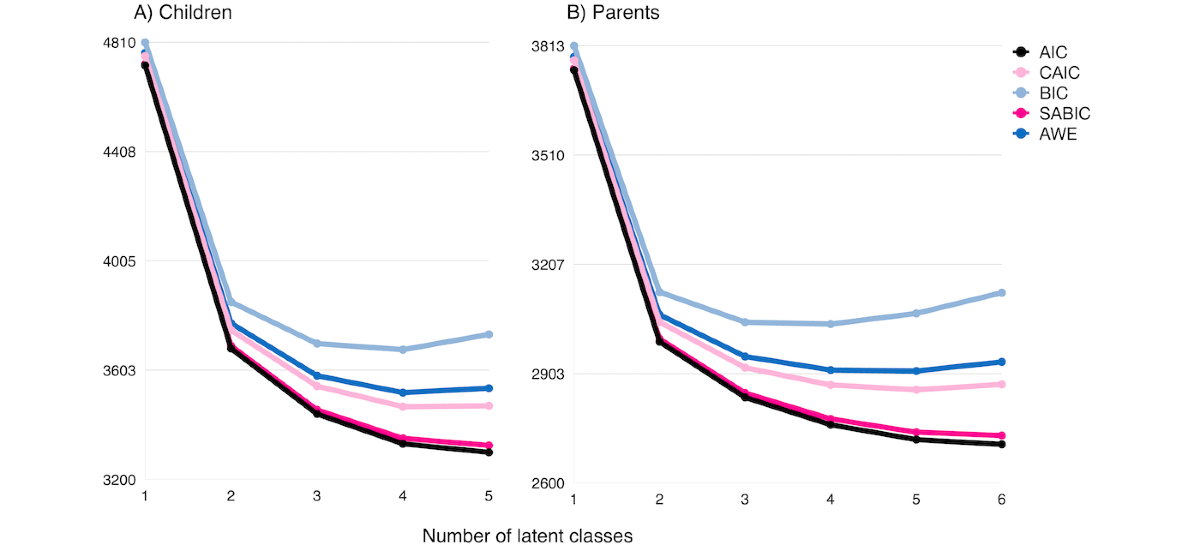
Plot of information criterion values across latent classes among children (A) and parents (B). AIC: Akaike information criterion; BIC: Bayesian information criterion; CAIC: consistent AIC; AWE: approximate weight of evidence; SABIC: sample size–adjusted BIC.

Table 1 summarizes the results from the LCA and Figure 4 provides plots for selecting LCA indices for both the child and parent models. Fit indices and interpretability of the classes supported a 4-class solution among children. By contrast, the parent LCA fit indices pointed to a 5- or 6-class solution; however, further evaluation of the potential solutions led to the retention of the 5-class solution. Although 6 of the 11 indices showed the 6-class solution as the best option, the relatively small sample for 2 of the classes suggested an overextraction. Therefore, the 5-class model was retained as the final solution for the parents. In addition, the 5-class solution made more substantive sense. Moreover, the average posterior probability for both child- and parent-selected models ranged between 91% and 99%, indicating well-differentiated classes for the 5-class solution. Thus, our results suggest excellent differentiation between the classes.

**Figure 5:**
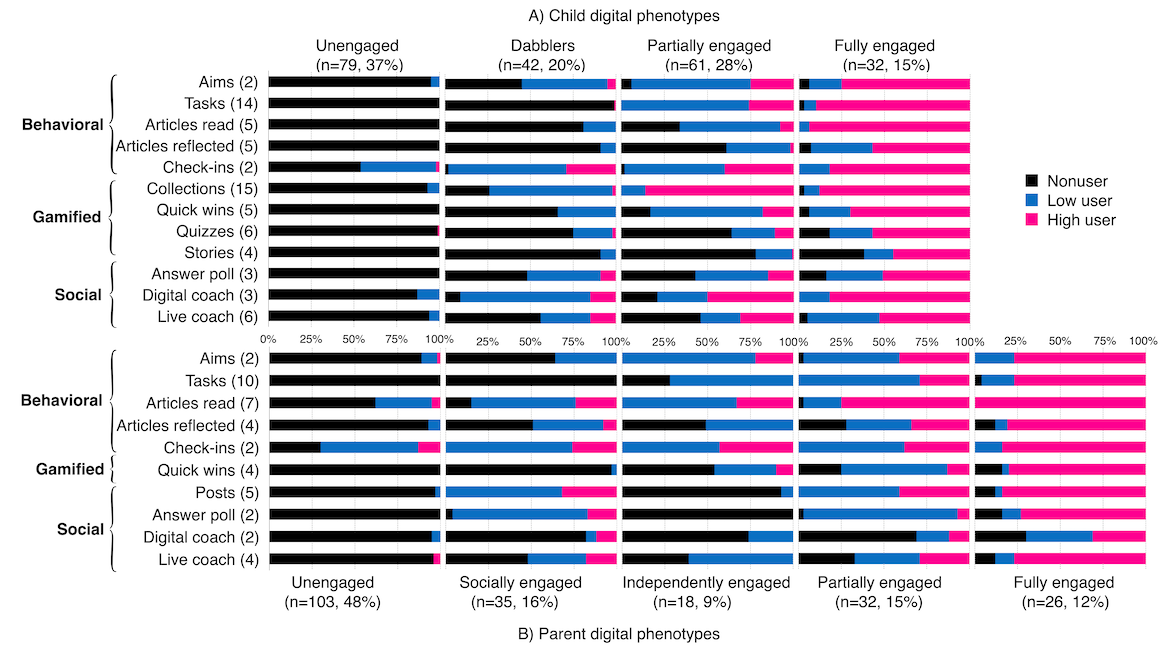
Conditional probability plots showing child (A) and parent (B) digital phenotypes (N=214). Numbers within brackets on the y-axis indicate the median distribution of use for each feature (eg, the median number of tasks completed by parents over 3 months was 10 among low and high users).

Figure 5 shows children’s and parents’ digital phenotypes (A and B, respectively). Figure 5A shows 4 children’s digital phenotypes (N=214): *unengaged*, *dabblers*, *partially engaged,* and *fully engaged*. *Unengaged* (79/214, 36.9% of users) included children who did not interact with most of the app features with exception of check-ins. *Dabblers* (42/214, 19.6% of users) regrouped children who did not use most behavioral features of the app (eg, completing tasks and reading or reflecting on articles) but predominantly interacted with gamified and social features, including collections and the digital coach. *Partially engaged* (61/214, 28.5% of users) included children who were low users of the behavioral features, particularly regarding task completion and reading and reflecting on articles but had greater use of the check-in feature. *Partially engaged* children had mixed interactions with the gamified and social features, with greater use of the collections and the digital coach, respectively, but rarely read stories or completed quizzes. *Fully engaged* (32/214, 15% of users) comprised high users of most app features and included children who engaged the most with the *active ingredients* of the app (ie, the behavioral features such as setting aims and completing tasks).

Figure 5B shows 5 parental digital phenotypes (N=214): *unengaged*, *socially engaged*, *independently engaged*, *partially engaged,* and *fully engaged*. *Unengaged* (103/214, 48.1% of users) included parents who did not use most of the features, with the exception of check-ins. *Socially engaged* (35/214, 16.4% of users) regrouped parents who engaged with the social features of the app by creating posts on the social wall, answering poll questions, and interacting with the live health coach. However, *socially engaged* parents had low use of the behavioral and gamified features and, in particular, did not complete any tasks within the app. *Independently engaged* (18/214, 8.4% of users) comprised parents who made little use of the social features (the only social feature they used involved direct messages with the live health coach but did not interact with other parents). Instead, *independently engaged* parents focused their attention on the behavioral features of the app and mostly set aims, read articles, and completed check-ins; however, they also interacted with all the behavioral features to some degree. *Partially engaged* (32/214, 15% of users) included parents who had a mixed use of most app features, indicating that their engagement with some behavioral (eg, aims and check-ins) and social (eg, posts) features was evenly split between low and high use. *Partially engaged* parents tended to be high users of the article feature, low users of the answer poll feature, and nonusers of the digital coach feature. Hence, their overall engagement with the behavioral features tended to be greater than with the gamified and social features. Finally, *fully engaged* parents (26/214, 12.1% of users) included users who interacted extensively with all app features, except the digital coach feature.

**Table 1:** Comparative fit indices between k-class solutions for children and parents.

Classes in the model^a^		LL^b^	AIC^c^	BIC^d^	SABIC^e^	VLMR-LRT^f^ *P* value	BLRT^g^ *P* value	Entropy^h^	CAIC^i^	AWE^j^	BF^k^	CmP^l^
**Children’s models**												
	1	−2337	4725	4809	4730	N/A^m^	N/A	N/A	4758	4771	0.0	0.0
	2	−1790	3682	3853	3692	<.001	<.001	.95	3750	3775	0.0	0.0
	3	−1644	3441	3700	3456	.10	<.001	.95	3544	3582	0.3	0.2
	4^n^	−1563	3331	3678	3351	.008	<.001	.96	3468	3520	16.6	0.8
	5^o^	−1521	3300	3734	3325	.76	<.001	.95	3471	3536	71.5	0.0
**Parents’ models**												
	1	−1853	3746	3813	3749	N/A	N/A	N/A	3772	3782	0.0	0.0
	2	−1455	2992	3130	3000	<.001	<.001	.95	3046	3067	0.0	0.0
	3	−1357	2837	3046	2850	.09	<.001	.93	2920	2951	0.8	0.4
	4	−1298	2761	3041	2778	.90	<.001	.97	2872	2913	4.4	0.5
	5^n^	−1256	2720	3070	2741	.12	<.001	.98	2859	2911	17.7	0.1
	6	−1229	2707	3128	2732	.79	.01	.99	2873	2936	22.7	0.0

^a^Model and number of classes in the solution.

^b^LL: log-likelihood.

^c^AIC: Akaike information criterion.

^d^BIC: Bayesian information criterion.

^e^SABIC: sample size–adjusted Bayesian information criterion.

^f^VLMR-LRT: Vuong-Lo-Mendell-Rubin adjusted likelihood ratio test.

^g^BLRT: bootstrapped likelihood ratio test.

^h^Entropy or differentiation between classes.

^i^CAIC: consistent Akaike information criterion.

^j^AWE: approximate weight of evidence.

^k^BF: Bayes factor.

^l^CmP: correct model probability.

^m^N/A: not applicable.

^n^Selected solution based on fit indices, relative sample sizes, and interpretability.

^o^This model was not identified, but the results are reported only for transparency purposes.

### Demographic Characteristics Associated With Digital Phenotypes

Table 2 and Table 3 show the distribution of demographic factors across child and parent digital phenotypes, respectively, with relative risk ratios and significance levels available in Multimedia Appendix 2 (Tables S1 and S2). The results are presented separately for children and parents. Children in the *fully engaged* phenotype were 1 to 1.5 years younger than children belonging to the *dabblers* (*P*=.04) and *unengaged* (*P*=.003) phenotypes. Furthermore, children from the *dabblers* phenotype were more likely to be in a household with an income >CAD $80,000 (US $63,771) than children belonging to the *fully* and *partially engaged* phenotypes (*P*=.03 and *P*=.047, respectively). Parents in the *socially engaged* phenotype were 2 to 3 years older than parents in the *fully engaged* (*P*=.02) and *unengaged* (*P*=.01) phenotypes. Moreover, *fully engaged* parents were more likely to be married, common law, or living with a partner than parents belonging to the *independently engaged* (*P*=.02*)* and *unengaged* (*P*=.01) phenotypes, who were more likely to be single, divorced, or widowed.

**Figure 6:**
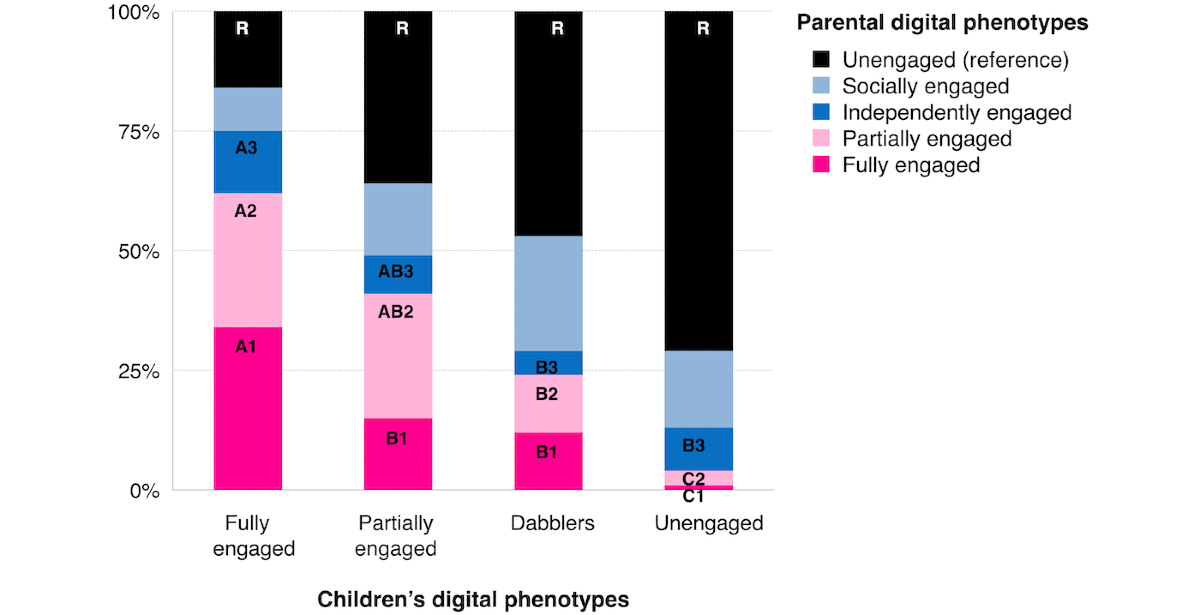
Associations between children’s and parents’ digital phenotypes (N=214). Vertical bars represent the proportion of parents with each phenotype with a given child phenotype. Within groups that share the same number and color, groups that do not share the same letter are significantly different from one another and are compared with the reference group (ie, unengaged users).

Figure 6 shows the distribution of parental digital phenotypes across children’s phenotypes, highlighting how their phenotypes were strongly associated. At one end of the spectrum, *fully engaged* children were more likely to have *fully* and *partially engaged* parents, and at the other end, *unengaged* children were more likely to have *unengaged* parents.

**Table 2:** Demographic distribution across child digital phenotypes (N=214).

Predictors of child digital phenotypes^a^	Fully engaged	Partially engaged	Dabblers	Unengaged
Phenotype sample size, N	32	61	42	79
Age (years), mean (SD)^b^	12.0 (1.8)	12.9 (2.3)	13.0 (2.4)	13.5 (2.2)
Sex (female), n (%)	19 (59)	30 (49)	19 (45)	42 (53)
Household income (≥CAD $80,000; US $63,771), n (%)^c^	17 (53)	31 (51)	29 (69)	42 (53)
Parental education (more than a Bachelor’s degree), n (%)	15 (47)	28 (46)	18 (43)	32 (41)
Parental marital status (married), n (%)	27 (84)	46 (75)	32 (76)	58 (73)
Race or ethnicity (White or European), n (%)	22 (69)	38 (62)	28 (67)	41 (52)

^a^Predictors’ reference groups are: male, household income <CAD $80,000 (US $63,771), parental educational attainment lower than a bachelor’s degree, single parents, and people who did not self-identify as having a White or European descent.

^b^The age of *fully engaged* children significantly differs from both *dabblers* and *unengaged* children’s age.

^c^The household income of both *fully engaged* and *partially engaged* children significantly differs from the household income among *dabblers*.

**Table 3:** Demographic distribution across parent digital phenotypes (N=214).

Predictors of parent digital phenotypes^a^	Fully engaged	Partially engaged	Independently engaged	Socially engaged	Unengaged
Phenotype sample size, N	26	32	18	35	103
Age (years), mean (SD)^b^	44.5 (7.1)	42.2 (5.6)	44.5 (7.1)	46.7 (6.6)	43.5 (6.0)
Sex (female), n (%)	26 (100)	32 (100)	17 (94.4)	31 (88.6)	92 (89.3)
Household income (≥CAD $80,000; US $63,771), n (%)	16 (61.5)	20 (62.5)	9 (50)	17 (48.6)	57 (55.3)
Parental education (more than a Bachelor’s degree), n (%)	9 (34.6)	20 (62.5)	9 (50)	14 (40)	41 (39.8)
Parental marital status (married, common law, or living with a partner), n (%)^c^	25 (96.2)	27 (84.4)	12 (66.7)	26 (74.3)	73 (70.9)
Race or ethnicity (White or European), n (%)	20 (76.9)	19 (59.3)	11 (61.1)	20 (57.1)	59 (57.3)
Recruitment through a clinical setting, n (%)	9 (34.6)	14 (43.8)	8 (44.4)	17 (48.6)	47 (45.6)

^a^Predictors’ reference groups are: male, household income <CAD $80,000 (US $63,771), parental educational attainment lower than a bachelor’s degree, single parents, people who did not self-identify as having a White or European descent, and recruitment through Facebook.

^b^The age of both *fully engaged* and *unengaged* parents significantly differs from the age of *socially engaged* parents.

^c^The marital status of *fully engaged* parents significantly differs from both *independently engaged* and *unengaged* parents’ marital status.

### Changes in Health Outcomes Across Digital Phenotypes

**Figure 7:**
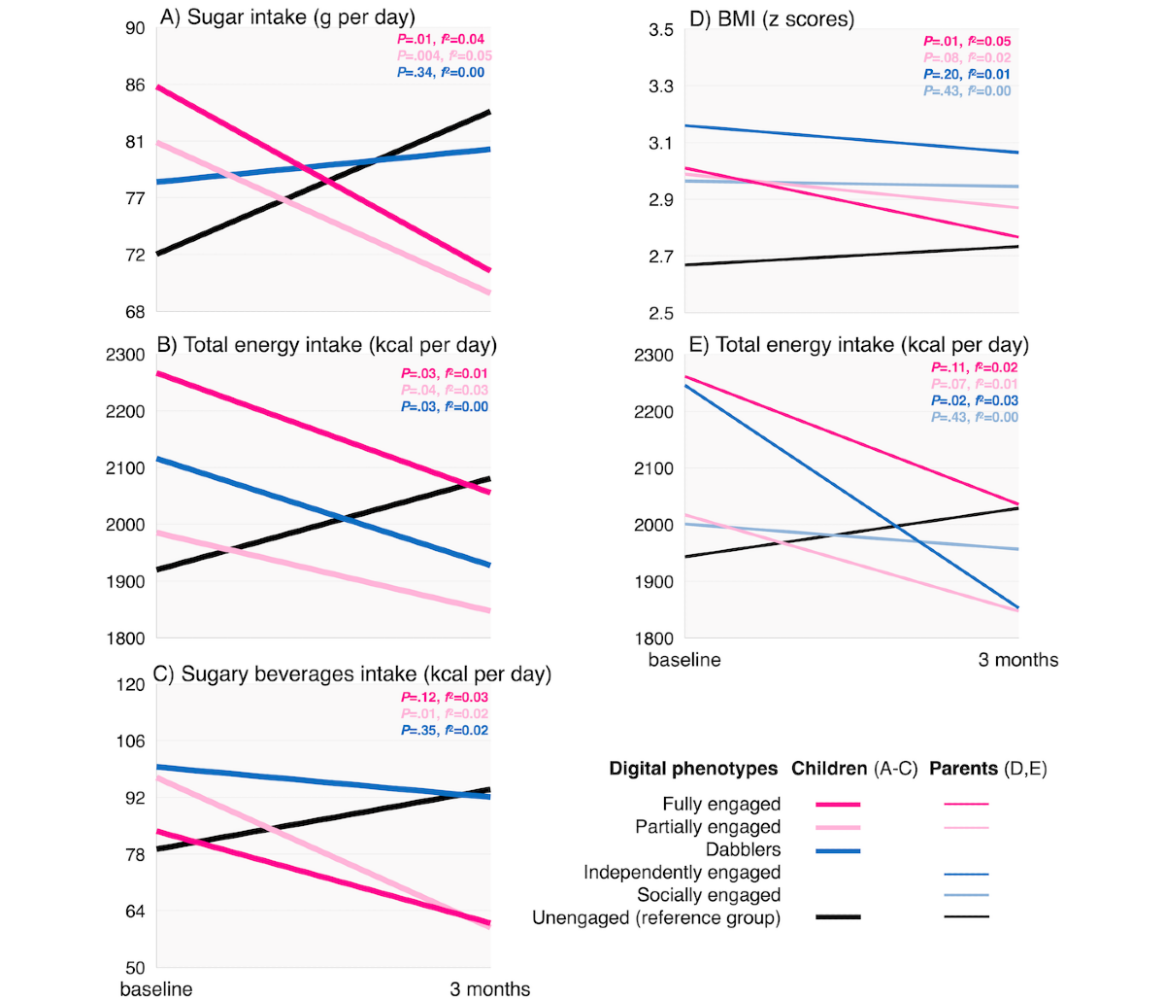
Changes in children’s health outcomes across children’s (A-C) and parents’ (D and E) digital phenotypes (N=214). Comparison of each phenotype versus unengaged phenotype (reference group). *P* value indicates significance level and f2 indicates Cohen effect size.

Table 4 summarizes the 3-month changes in *z*BMI, diet, physical activity, and screen time across children’s and parents’ digital phenotypes, with statistically significant (*P*<.05) or borderline significant (*P<*.10) comparisons shown in Figure 7, where panels A to C show child outcomes across child phenotypes, and panels D and E show child outcomes across parent phenotypes.

Multiple group comparisons showed that changes in the total sugar intake of children differed across phenotypes (*P*=.01; Figure 7A-C). Children belonging to the *fully engaged* (*P*=.01; *f*^2^=0.04) or *partially engaged* (*P*=.004; *f*^2^=0.05) phenotypes reduced their total sugar intake over 3 months compared with children in the *unengaged* phenotype (reference group), who increased their total sugar intake over time. Regarding children’s total daily energy intake and energy intake from sugary beverages, we found borderline differences (*P*=.07 and *P*=.09) that became significant in individual pairwise comparisons. Children from the *fully engaged* (*P*=.03; *f*^2^=0.01), *partially engaged* (*P*=.04; *f*^2^=0.03), and *dabblers* (*P*=.03; *f*^2^=0.00) phenotypes decreased their total energy intake over 3 months compared with the *unengaged* children who increased their daily energy intake over time. Finally, children from the *partially engaged* phenotype decreased their intake of sugary beverages compared with *unengaged* children who did not (*P*=.01; *f*^2^=0.02). In this case, *fully engaged* children did not differ significantly from *unengaged* children; however, as shown in Figure 7C, children’s intake of sugary beverages in the *fully engaged* group trended downward, whereas *unengaged* children’s intake trended upward (*P*=.12).

Differential changes in outcomes among children were also observed across the parental phenotypes (Figure 7D and 7E). Multiple group comparisons showed borderline significant changes in children’s *z*BMI and total daily energy intake across parental phenotypes (*P*=.06 and *P*=.08, respectively), which became significant in individual pairwise comparisons. Specifically, children whose parents were *fully engaged* significantly decreased their *z*BMI (*P*=.01; *f*^2^=0.05) compared with children with *unengaged* parents (reference group) whose *z*BMI slightly increased. Similarly, children whose parents belonged to the *independently engaged* phenotype decreased their daily caloric intake (*P*=.02; *f*^2^=0.03) compared with children with *unengaged* parents whose daily caloric intake increased over 3 months. Figure 7E also shows trends of decreased caloric intake among children with *fully* and *partially engaged* parents compared with children with *unengaged* parents; however, these trends were not statistically significant (*P*=.11 and *P*=.07, respectively).

**Table 4:** Changes in children’s and parents’ health outcomes across digital phenotypes (N=214).

Participants and health outcomes			Child phenotypes					Parent phenotypes		
			Chi-square (*df*)		*P* value		Chi-square (*df*)			*P* value
**Children**										
	BMI *z* scores	0.5 (3)		.93		9.1 (4)^a^			.06^a^	
	Total energy, daily (kcal per day)	7.2 (3)^a^		.07^a^		8.2 (4)^a^			.08^a^	
	Healthy Eating Index (range 0-100 points)	0.3 (3)		.96		1.9 (4)			.76	
	Fruits and vegetables (daily servings)	0.2 (3)		.98		3.6 (4)			.47	
	Saturated and trans fat (g per day)	5.4 (3)		.15		5.5 (4)			.24	
	Total fiber (g per day)	1.5 (3)		.68		1.5 (4)			.84	
	Total sugar (g per day)	11.8 (3)^a^		.01^a^		5.4 (4)			.25	
	Sugary beverages (kcal per day)	6.7 (3)^a^		.09^a^		5.5 (4)			.24	
	Total physical activity (minutes per week)	2.5 (3)		.47		0.6 (4)			.96	
	Fitbit (steps per day)	2.1 (3)		.55		3.2 (4)			.52	
	Screen time (minutes per day)	4.9 (3)		.18		2.3 (4)			.69	
**Parents**										
	Daily frequency of sugary beverages (times per day)	N/A^b^		N/A		1.2 (4)			.88	
	Daily frequency of fruit juice (times per day)	N/A		N/A		6.5 (4)			.16	
	Fruit and vegetables (servings per day)	N/A		N/A		2.4 (4)			.67	
	Walking (minutes per day)	N/A		N/A		4.0 (4)			.41	
	Moderate and vigorous physical activity (minutes per day)	N/A		N/A		2.0 (4)			.75	
	Screen time (minutes per week)	N/A		N/A		7.1 (4)			.13	

^a^Indicate significant (*P<*.05) or borderline significant (*P<*.10) interactions (time×digital phenotype) for which pairwise comparisons between phenotypes were further explored.

^b^N/A: not applicable.

## Discussion

### Principal Findings

This is the first digital phenotyping study of an mHealth intervention targeting health behavior changes among children with overweight or obesity and their parents. We evaluated user typologies based on how children and parents interacted with different features of the Aim2Be app. We found 4 child (*unengaged, dabblers, partially engaged,* and *fully engaged*) and 5 parent (*unengaged, socially, independent, partially engaged,* and *fully engaged*) phenotypes, which illustrate the ways in which participants used the behavioral, gamified, and social features of the Aim2Be app. As expected, based on Aim2Be’s conceptual framework [25], our results demonstrated that specific patterns of use supported behavior change, whereas others did not, meaning that greater engagement with the active ingredients of the app improved children’s dietary and weight outcomes.

### Comparison With Prior Work

Given the scarcity of research on the digital phenotypes of mHealth users in the context of childhood obesity, it is difficult to compare our findings with those of previous studies. However, our results are similar to a recently published study profiling children’s (but not parents’) engagement with an older version of Aim2Be [33], where the 4 child profiles that emerged were similar, although our study examined 6 additional app features. Interestingly, the results previously observed in the prevention context [33] were replicated in our study using a clinical sample of children. Importantly, users with distinct patterns of engagement obtained different health benefits depending on whether they interacted with the active ingredients of the app. When lifestyle behavior modification interventions required in-person attendance, dose-response studies identified a minimum of 26 hours of contact for the intervention to improve children’s outcomes [4]. However, our digital phenotype analyses illustrate that new approaches are needed to conduct dose-response analyses in the context of mHealth interventions, especially when users have the freedom to select which app features they engage with. As users interact with the Aim2Be app quite differently, this variability must be accounted for when assessing whether the intervention can influence the mediators and outcomes targeted by the app.

In this study, we found that *fully engaged* children with Aim2Be (eg, set goals, completed tasks, and read articles) experienced more desirable behavior changes than *unengaged* users. Specifically, children who engaged more fully with the app decreased their intake of total daily calories, total sugars, and sugary beverages. Our findings align with existing research [22,33] suggesting that mHealth interventions have the potential to improve children’s dietary behaviors. Furthermore, in exploratory analyses examining the aims that were most often set and completed among Aim2Be users (data not shown), we found that “Drop sugary drinks” was the most common aim chosen by children, which validates our findings related to lower total sugar and energy from sugary drinks among *fully* and *partially engaged* children. These results highlight the importance of increasing engagement with the app’s *active ingredients*, namely, setting specific goals and completing tasks related to those goals to promote health behavior change among children.

In this study, *fully engaged* children were more likely to be younger and have *fully* or *partially engaged* parents. These associations could indicate that the app was more appealing to younger children, as shown by other research [34], or that parents dedicated more attention to their children when they were younger than when they were older. In addition, younger children might be more easily influenced by their parents, which may explain their use of the Aim2Be app. These findings are aligned with previous studies reporting that parental self-monitoring (a behavioral strategy) and adherence to eHealth interventions were significant predictors of adolescents’ self-monitoring and adherence [33,64].

We also found that children whose parents were *fully* or *partially engaged* with the app’s behavioral features decreased their *z*BMI and total daily energy intake more than children whose parents only engaged with the social features or who did not engage with the app at all. Our findings are consistent with a qualitative study [65] showing that participation *as a family* is one of the main factors identified by both children and parents to facilitate behavior change. Indeed, current guidelines [4,15,66] for the treatment of childhood obesity include a family-based approach. Taken together, our findings reinforce the critical role that parents play in lifestyle interventions to support their children’s adherence and improvement of health outcomes, even in the mHealth context.

This study also revealed that family structure was associated with parental phenotypes. Fewer single-parent households belonged to the *fully* and *partially engaged* phenotypes than parents who lived with a partner or were married, which may reflect that more independent, time-scarce (and therefore task-oriented) parents [67]. Interestingly, single-parent households were also likely to belong to the *independently engaged* phenotype (ie, parents who only engaged with the behavioral app features such as aims and tasks), and children whose parents belonged to this phenotype reduced more of their total daily energy than other phenotypes. In fact, previous research found that parents of young children decreased their use of mHealth apps when they had limited time or only used the app to find specific information of interest [67]. This could explain why *independently engaged* parents did not use the gamified or social domains but used the domain exclusively focused on behavioral change and why their children decreased their energy intake over time.

### Limitations and Strengths

This study had several limitations and strengths. First, our sample was relatively small and not powered to detect significant changes across multiple digital phenotypes in these secondary analyses. This could have limited our ability to detect clinically meaningful changes in health outcomes, although some changes were observed. In addition, overall adherence to the app was low, which limited our ability to detect more phenotypes and perhaps to observe some between-group changes. Moreover, our study included a clinical sample (children with overweight or obesity); thus, our findings are limited to this population. Nevertheless, we used a detailed dietary assessment (24-hour dietary recalls), both self-reported and objective measures of physical activity, and objective measures of app usability through app analytics. Finally, we used a novel approach to examine intervention efficacy, which showed positive effects that are not observed [38] using more traditional analysis.

### Future Directions

Overall, 3 key messages from our findings point to future directions in mHealth research. First, even in the mHealth context, parental engagement matters as it can increase children’s adherence to a lifestyle intervention and provide the household environment that supports behavior change. Thus, whether a lifestyle intervention is delivered in person or on the web, parents should be involved as they are active agents of change. Second, dose-response analyses should assess how (and not only how much) the app is being used by the participants, as users’ full engagement with the *active ingredients* of the app seems to be a critical factor for the success of mHealth interventions. Finally, as participants’ engagement with specific features of an app is key to promoting behavior change, future research should examine how we design program components that ensure users interact with the active ingredients of the mHealth intervention.

### Conclusions

This study showed that distinct patterns of use exist among both parents and children who used a family-based lifestyle mHealth app, namely, Aim2Be. Identifying who uses mHealth apps and how can help us understand and develop more tailored interventions to support various users in a health behavior change process. Our findings point to the importance of optimizing users’ full engagement with the *active ingredients* of the app as a critical factor for the success of mHealth interventions and highlight the need for further research to understand program design elements that can influence participant engagement.

## Appendix

Multimedia Appendix 1 CONSORT-EHEALTH (Consolidated Standards of Reporting Trials of Electronic and Mobile Health Applications and Online Telehealth) checklist.

Multimedia Appendix 2 Relative risk ratios of the predictors of the digital phenotypes.

## Abbreviations

CONSORT-EHEALTH: Consolidated Standards of Reporting Trials of Electronic and Mobile Health Applications and Online Telehealth
LCA: latent class analysis
mHealth: mobile health
zBMI: BMI z score

## Data Availability

LCM should be contacted at the BC Children’s Hospital Research Institute and School of Population and Public Health University of British Columbia (lmasse@bcchr.ubc.ca) regarding access to data and materials.
