# Supplementary material for: The Aim2Be mHealth Intervention for Children With Overweight or Obesity and Their Parents: Person-Centered Analyses to Uncover Digital Phenotypes
Source: J Med Internet Res. 2022 Jun 22;24(6):e35285. doi: 10.2196/35285 (PMC9221987; doi:10.2196/35285)
Supplement: Multimedia Appendix 2 [file jmir_v24i6e35285_app2.docx]

# The Aim2Be mHealth Intervention for Children With Overweight or Obesity and Their Parents: Person-Centered Analyses to Uncover Digital Phenotypes

Olivia De-Jongh González, Claire N. Tugault-Lafleur, E. Jean Buckler, Jill Hamilton, Josephine Ho, Annick Buchholz, Katherine M. Morrison, Geoff D.C. Ball and Louise C. Mâsse

# Multimedia Appendix: Relative Risk Ratios of the predictors of the digital phenotypes of the Aim2Be mHealth users

**Table S1.** Predictors of children’s digital phenotypes (n=214)

|  | Reference phenotype | | | |
| --- | --- | --- | --- | --- |
|  | Fully engaged | Partially engaged | Dabblers | Unengaged |
| Comparison phenotype ^a^ | RRR ^b^ | RRR | RRR | RRR |
|  |  |  |  |  |
| **Fully engaged (n=32, 15%)** |  |  |  |  |
| Child’s age |  | 0.80 | 0.77^c^ | 0.69^d^ |
| Child’s sex: female |  | 1.80 | 1.94 | 1.34 |
| Household income: > $CAD 80,000 |  | 0.71 | 0.23^c^ | 0.44 |
| Parental education: > bachelor’s degree |  | 1.29 | 1.41 | 1.32 |
| Parental marital status: married |  | 1.17 | 1.65 | 1.04 |
| Race/ethnicity: white |  | 1.08 | 0.85 | 1.59 |
| Parental digital phenotype |  |  |  |  |
| Fully engaged |  | 5.70^c^ | 10.87^d^ | 157.95^e^ |
| Partially engaged |  | 2.14 | 6.43^c^ | 50.19^e^ |
| Independently engaged |  | 3.87 | 8.24^c^ | 7.08^c^ |
| Socially engaged |  | 1.66 | 1.39 | 3.33 |
| **Partially engaged (n=61, 28%)** |  |  |  |  |
| Child’s age | 1.25 |  | 0.96 | 0.86 |
| Child’s sex: female | 0.55 |  | 1.07 | 0.74 |
| Household income: > $CAD 80,000 | 1.42 |  | 0.33^c^ | 0.62 |
| Parental education: > bachelor’s degree | 0.77 |  | 1.09 | 1.02 |
| Parental marital status: married | 0.86 |  | 1.41 | 0.89 |
| Race/ethnicity: white | 0.93 |  | 0.79 | 1.48 |
| Parental digital phenotype |  |  |  |  |
| Fully engaged | 0.18^c^ |  | 1.91 | 27.73^d^ |
| Partially engaged | 0.47 |  | 3.01 | 23.50^e^ |
| Independently engaged | 0.26 |  | 2.13 | 1.83 |
| Socially engaged | 0.60 |  | 0.84 | 2.01 |
| **Dabblers (n=42, 20%)** |  |  |  |  |
| Child’s age | 1.30^c^ | 1.04 |  | 0.89 |
| Child’s sex: female | 0.52 | 0.93 |  | 0.69 |
| Household income: > $CAD 80,000 | 4.31^c^ | 3.05^c^ |  | 1.90 |
| Parental education: > bachelor’s degree | 0.71 | 0.91 |  | 0.93 |
| Parental marital status: married | 0.61 | 0.71 |  | 0.63 |
| Race/ethnicity: white | 1.17 | 1.27 |  | 1.87 |
| Parental digital phenotype |  |  |  |  |
| Fully engaged | 0.09^d^ | 0.52 |  | 14.53^c^ |
| Partially engaged | 0.16^c^ | 0.33 |  | 7.80^c^ |
| Independently engaged | 0.12^c^ | 0.47 |  | 0.86 |
| Socially engaged | 0.72 | 1.19 |  | 2.40 |
| **Unengaged (n=79, 37%)** |  |  |  |  |
| Child’s age | 1.46^d^ | 1.17 | 1.12 |  |
| Child’s sex: female | 0.75 | 1.35 | 1.45 |  |
| Household income: > $CAD 80,000 | 2.27 | 1.60 | 0.53 |  |
| Parental education: > bachelor’s degree | 0.76 | 0.98 | 1.07 |  |
| Parental marital status: married | 0.96 | 1.12 | 1.58 |  |
| Race/ethnicity: white | 0.63 | 0.68 | 0.54 |  |
| Parental digital phenotype |  |  |  |  |
| Fully engaged | 0.01^e^ | 0.04^d^ | 0.07^c^ |  |
| Partially engaged | 0.02^e^ | 0.04^e^ | 0.13^c^ |  |
| Independently engaged | 0.14^c^ | 0.55 | 1.16 |  |
| Socially engaged | 0.30 | 0.50 | 0.42 |  |

^a^ Predictors’ reference groups are: male; household income < $CAD 80,000; parental education of college degree or below; single parents, race/ethnicity other than White; and parental *Unengaged* phenotype.

^b^ RRR: Relative risk ratios of the outcome falling in the comparison phenotype (left column) compared to the reference phenotypes (top of the table).

**^c^** *P*<.05

**^d^** *P*≤.01

**^e^** *P*≤.001

**Table S2.** Predictors of parents’ digital phenotypes (n=214)

|  | Reference phenotype (n=214) | | | | |
| --- | --- | --- | --- | --- | --- |
|  | Fully engaged | Partially engaged | Independently engaged | Socially engaged | Unengaged |
| Comparison phenotype ^a^ | RRR ^b^ | RRR | RRR | RRR | RRR |
|  |  |  |  |  |  |
| **Fully engaged (n=26, 12%)** | | | | | |
| Parents’ age |  | 0.95 | 0.94 | 0.89^c^ | 0.98 |
| Household income: > $CAD 80,000 |  | 0.82 | 1.04 | 1.01 | 0.58 |
| Parental education: >bachelor’s degree |  | 0.35 | 0.47 | 0.97 | 0.85 |
| Parental marital status: married |  | 8.41 | 19.35^c^ | 9.89 | 16.42^c^ |
| Race/ethnicity: White |  | 2.56 | 2.51 | 2.34 | 2.61 |
| Recruitment through clinical settings |  | 0.92 | 0.84 | 1.18 | 0.94 |
| **Partially engaged (n=32, 15%)** | | | | | |
| Parents’ age | 1.05 |  | 0.99 | 0.94 | 1.03 |
| Household income: > $CAD 80,000 | 1.21 |  | 1.26 | 1.22 | 0.70 |
| Parental education: >bachelor’s degree | 2.83 |  | 1.34 | 2.74 | 2.41 |
| Parental marital status: married | 0.12 |  | 2.30 | 1.18 | 1.95 |
| Race/ethnicity: white | 0.39 |  | 0.98 | 0.92 | 1.02 |
| Recruitment through clinical settings | 1.09 |  | 0.91 | 1.28 | 1.02 |
| **Independently engaged (n=18, 9%)** | | | | | |
| Parents’ age | 1.06 | 1.01 |  | 0.94 | 1.04 |
| Household income: > $CAD 80,000 | 0.97 | 0.80 |  | 0.97 | 0.56 |
| Parental education: >bachelor’s degree | 2.11 | 0.74 |  | 2.04 | 1.79 |
| Parental marital status: married | 0.05^c^ | 0.43 |  | 0.51 | 0.85 |
| Race/ethnicity: white | 0.40 | 1.02 |  | 0.93 | 1.04 |
| Recruitment through clinical settings | 1.19 | 1.10 |  | 1.41 | 1.12 |
| **Socially engaged (n=35, 16%)** | | | | | |
| Parents’ age | 1.12^c^ | 1.07 | 1.06 |  | 1.10^d^ |
| Household income: > $CAD 80,000 | 0.99 | 0.82 | 1.03 |  | 0.57 |
| Parental education: >bachelor’s degree | 1.03 | 0.37 | 0.49 |  | 0.88 |
| Parental marital status: married | 0.10 | 0.85 | 1.96 |  | 1.66 |
| Race/ethnicity: white | 0.43 | 1.09 | 1.07 |  | 1.11 |
| Recruitment through clinical settings | 0.85 | 0.78 | 0.71 |  | 0.80 |
| **Unengaged (n=103, 48%)** | | | | | |
| Parents’ age | 1.02 | 0.97 | 0.97 | 0.91^d^ |  |
| Household income: > $CAD 80,000 | 1.73 | 1.43 | 1.79 | 1.75 |  |
| Parental education: >bachelor’s degree | 1.17 | 0.42 | 0.56 | 1.14 |  |
| Parental marital status: married | 0.06^c^ | 0.51 | 1.18 | 0.60 |  |
| Race/ethnicity: White | 0.38 | 0.98 | 0.96 | 0.90 |  |
| Recruitment through clinical settings | 1.06 | 0.98 | 0.89 | 1.25 |  |

^a^ Predictors’ reference groups were: male; household income < $CAD 80,000; parental education of college degree or below; single parents, race/ethnicity other than White; and recruitment through Facebook.

^b^ RRR: Relative risk ratios of the outcome falling in the comparison phenotype (left column) compared to the reference phenotype (top of the table).

**^c^** *P*<.05

**^d^** *P*≤.01
